# Supplementary material for: Role of VapBC4 toxin-antitoxin system of Sulfolobus acidocaldarius in heat stress adaptation
Source: mBio. 2024 Nov 13;15(12):e02753-24. doi: 10.1128/mbio.02753-24 (PMC11633383; doi:10.1128/mbio.02753-24)

**Supplementary Information**

Role of VapBC4 toxin-antitoxin system of *Sulfolobus acidocaldarius* in the heat stress adaptation

Arghya Bhowmick^1^, Alejandra Recalde^2^, Chandrima Bhattacharyya^1^, Ankita Banerjee^1^, Jagriti Das^1^, Ulises E. Rodriguez-Cruz^3^, Sonja-Verena Albers^2^, Abhrajyoti Ghosh^1^

^1^ Department of Biological Sciences, Bose Institute, EN Block, Sector V, Kolkata-700091, India

^2^ Molecular Biology of Archaea, Microbiology, Faculty of Biology, University of Freiburg, Freiburg, Germany

^3^ Department of Evolutionary Ecology, Institute of Ecology, National Autonomous University of Mexico, Mexico City, Mexico

***Correspondence:** A. Ghosh, Department of Biological Sciences, Bose Institute, Unified Academic Campus, EN 80, Sector V, Bidhan Nagar, Kolkata- 700091, West Bengal, India

Tel: +91-33-2569-3284; E-mails: abhrajyoti.ghosh@jcbose.ac.in; [aghosh78@gmail.com](mailto:aghosh78@gmail.com)

**Supplementary tables**

**Table S1:** List of primers used in the present study

**Table S2:** Strains used in the study

**Table S3:** Plasmids used in the study

**Table S4:** The list of organisms whose genomes were used to perform phylogenomic analysis

**Table S5:** Description of the putative 25 pairs homologous genes of the toxin-antitoxin system

**Supplementary figures**

**Figure S1:** Phylogenetic perspective of the 25 homologues of the VapB4 (Saci_1812). The leaves of the tree that correspond to lineages of organisms. The leaves of the tree that corresponds to lineage of the genus *Sulfolobus acidocaldarius* are highlighted in blue

**Figure S2:** Phylogenetic perspective of the 25 homologues of the VapC4 (Saci_1813). The leaves of the tree correspond to lineages of organisms. The leaves of the tree that corresponds to lineage of the genus *Sulfolobus acidocaldarius* are highlighted in blue

**Figure S3:** Western blot analysis of the Elution 2 fraction obtained after Ni-NTA affinity chromatography of VapC4 and VapB4 proteins using an anti-His antibody.

**Figure S4:** Graph showing the percentage of RNA following densitometric analysis of 23S rRNA and 16S rRNA bands from total RNA extracted from *Sulfolobus acidocaldarius* after exposure to increasing concentrations of the VapC4 toxin protein at two different temperatures (37°C and 60°C). The statistical significance, P < 0.05, is indicated as *, P < 0.01 is indicated as **, and P < 0.001 is indicated as ***; n.s. denotes not significant.

**Figure S5:** 1% Agarose gel showing RNase activity of VapC4 on different RNA substrates: a) tRNA^Ser^ b) tRNA^Pro^ c) *saci_1223* mRNA transcript and *saci_1242* mRNA transcript.

**Figure S6:** A 15% SDS-PAGE analysis revealed the absence of VapB4 antitoxin elution (at the 9 kDa position) in the Elution fraction when *E. coli* cells expressing solely the VapB4 antitoxin with a Strep-II tag underwent Ni-NTA affinity chromatography

**Figure S7**: Agarose gel showing PCR amplification products to verify the absence of *vapC4* and *vapBC4* genes in the Δ*vapC4* and Δ*vapBC4* strains of *S. acidocaldarius* MW2000 and to check the status of *vapC4*, *vapB4* and *vapBC4* genes in the generated complementation strain using *vapC4*, *vapB4* and *vapBC4* gene-specific primers, respectively

**Table S1:** List of primers used in the present study

| **Plasmids** | **Relevant characteristics** | **Source of reference** |
| --- | --- | --- |
| 12 | 5’-CCCCCGGTACCATGGTCAGGTAGGG-3’ Forward primer for Saci 7S SRP RNA with KpnI for cloning in pGEM3z vector | The present study |
| 13 | 5’-CCCCCAAGCTTGTCAGCTCCCCTATG-3’ Reverse primer for Saci 7S SRP RNA with HindIII for cloning into pGEM3z vector | The present study |
| 18 | 5’-ATGCGTCCGGCGTAGAG-3’ Forward sequencing primer for MCS1 of pETDuet1 | The present study |
| 19 | 5’-GATTATGCGGCCGTGTACAA-3’ Reverse sequencing primer for MCS1 of pETDuet1 | The present study |
| 20 | 5’-TTGTACACGGCCGCATAATC-3’ Forward sequencing primer for MCS2 of pETDuet1 | The present study |
| 21 | 5’- GCTAGTTATTGCTCAGCGG-3’ Reverse sequencing primer for MCS2 of pETDuet1 | The present study |
| 50 | 5’-TAATACGACTCACTATAGGG-3’ Forward sequencing primer for T7 promoter | The present study |
| 51 | 5’-GCTAGTTATTGCTCAGCGG-3’ Reverse sequencing primer for T7 promoter | The present study |
| 464 | 5’-CCCCCGGTACCTTCCGGTTGATCCYGCCGGA-3’ Forward primer for archaeal 16S rRNA gene with KpnI for cloning in pGEM3z vector | The present study |
| 465 | 5’-CCCCCAAGCTTYCCGGCGTTGAMTCCAATT-3’  Reverse primer for archaeal 16S rRNA gene with HindIII for cloning in pGEM3z vector | The present study |
| 562 | 5’-GGGGCCATGGGCATGACCGAGTACCTCTTTG-3’ Forward primer for *saci_1813* with NcoI for cloning in pET28a vector | The present study |
| 563 | 5’-GGGGCTCGAGTTGGTCAACGAATCCG-3’ Reverse primer for *saci_1813* with XhoI for cloning in pET28a vector | The present study |
| 564 | 5’-GGGGCCATGGGCATGGATAGCAGTGGATATG-3’ Forward primer for saci_1812 with NcoI for cloning in pET28a | The present study |
| 565 | 5’-GGGGCTCGAGACGACTTTCCCTATCTTCAC-3’ Reverse primer for *saci_1812* with XhoI for cloning in pET28a | The present study |
| 556 | 5’-CCCCGGTACCAGCGGCGTAGG-3’ Forward primer for *saci-tRNA^Met^* gene with KpnI for cloning in pGEM3z vector | The present study |
| 557 | 5’-CCCCAAGCTTTGGTAGCGGCGCCTG-3’ Reverse primer for *saci-tRNA^Met^* gene with HindIII for cloning in pGEM3z vector | The present study |
| 576 | 5’-GGGGCATATGATGGATAGCAGTGGATATG-3’ Forward primer for *saci_1812* with NdeI for cloning in MCS2 of pETDuet1 vector | The present study |
| 577 | 5’GGGGCTCGAGTCATTTTTCGAACTGGGGATGGC  TCCACCCACGACTTTCCCTATCTTCAC-3’ Reverse primer for *saci_1812* with XhoI for cloning in MCS2 of pETDuet1 vector (C terminal StrepII tag) | The present study |
| 579 | 5’-CCCCGGTACCAGGAAAAGAAATCAATTG-3’ Forward primer for *saci-23S rRNA* gene with KpnI for cloning in pGEM3z vector | The present study |
| 580 | 5’-CCCCAAGCTTTATTAGGCCCTACCTTAG-3’ Reverse primer for *saci-23S rRNA* gene with HindIII for cloning in pGEM3z vector | The present study |
| 581 | 5’-GGGGGGTACCATGTATAGGGCTCCTAAAAC-3’ Forward primer for *saci-tetR* with KpnI for cloning in pGEM3z vector | The present study |
| 582 | 5’-GGGGAAGCTTTTACCTTGGTGTTAAC-3’ Reverse primer for *saci-tetR* with HindIII for cloning in pGEM3z vector | The present study |
| 769 | 5’-CTTCCGCGGTTTCATCTTTCTGTAACC-3’ Forward primer for *vapB4* gene (*saci_1812*) including upstream 150 bps promoter with SacII for cloning in pSVA-ara-FX-stop (*vapB4* complementation) | The present study |
| 770 | 5’-CTTGTCGACTCAACGACTTTCCCTATC-3’ Reverse primer for *vapB4* gene (*saci_1812*) including upstream 150 bps promoter with SalI for cloning in pSVA-ara-FX-stop (*vapB4* complementation) | The present study |
| 771 | 5’-CTTGTCGACTCATTGGTCAACGAATCC-3’ Reverse primer for *vapBC4* operon (saci_1812/saci 1813) including 150 bps promoter with SalI for cloning in pSVA-ara-FX-stop (*vapBC4* complementation) | The present study |
| 772 | 5’-GTTAAAGGAATTGATGGACTAATATGG-3’ Forward primer for Site-directed mutagenesis to generate premature stop codon in the *vapB4* (*saci_1812*) gene (*vapC4* complementation) | The present study |
| 773 | 5’-CAGGTTTTATACCATATTAGTCCATC-3’ Reverse primer for Site-directed mutagenesis to generate premature stop codon in the *vapB4* (*saci_1812*) gene (*vapC4* complementation) | The present study |
| 801 | 5’- CCCCGGTACCGCCGGGGTCGCC-3’  Forward primer for *saci-tRNA^Ser^* gene with KpnI for cloning into pGEM3z vector | The present study |
| 802 | 5’- CCCCAAGCTTTGGCGCCGGGGG-3’  Reverse primer for *saci-tRNA^Ser^* gene with HindIII for cloning into pGEM3z vector | The present study |
| 867 | 5’-CCCCGGTACCATGGAGACAGTACTTCAAATTC-3’  Forward primer for *saci_1223* gene with KpnI for cloning into pGEM3z vector | The present study |
| 868 | 5’- CCCCGTCGACTTAAGACTTAAAAATATACTCT  TTTAAC-3’  Reverse primer for *saci_1223* gene with SalI for cloning into pGEM3z vector | The present study |
| 869 | 5’- CCCCGGTACCGTGAGCCAAGAAAAAATGGG-3’  Forward primer for *saci_1242* gene with KpnI for cloning into pGEM3z vector | The present study |
| 870 | 5’- CCCCAAGCTTCTATATCCTAGCCATATCCTCAC-3’  Reverse primer for *saci_1242* gene with HindIII for cloning into pGEM3z vector | The present study |
| 875 | 5’-CCCCGGTACCGGGGCCGTAGTCTAGCTTGG-3’  Forward primer for *saci-tRNA^Pro^* gene with KpnI for cloning into pGEM3z vector | The present study |
| 876 | 5’- CCCCAAGCTTTGGGGCCGCCGGGATTTG-3’  Reverse primer for *saci-tRNA^Pro^* gene with HindIII for cloning into pGEM3z vector | The present study |
| 13299 | 5’-GACTGGATCCCAACTAAGGCTATAATAAAGTAAGC-3’ Forward primer to amplify the upstream region of the *vapBC4* operon (*saci_1812/saci_1813*) (BamHI) | The present study |
| 14041 | 5’-TATTTTATGGATAGCCAATGACCGTAGAGACAAG AAC-3’ Reverse overlap primer to amplify the upstream region of the *vapBC4* operon (*saci_1812/saci_1813*) | The present study |
| 14042 | 5’-TCTCTACGGTCATTGGCTATCCATAAAATAATTTA TATG-3’ Forward overlap primer to amplify the downstream region of the *vapBC4* operon (*saci_1812/saci_1813*) | The present study |
| 13902 | 5’-GGGAATTCCATATGCAGGTTGTTGGAGAGAAGTAT GTC-3’ Reverse primer to amplify the downstream region of the *vapBC4* operon (*saci_1812/saci_1813*) (NdeI) | The present study |
| 13906 | 5’-GACTGGATCCCAGACCTTAAACACGTGAGAATTAG-3’ Forward primer to amplify the upstream region of the *vapC4* (*saci_1813*) gene (BamHI) | The present study |
| 13907 | 5’-TCTTGTCTCTACGGTCAACGACTTTCCCTATCTT CACGTA-3’ Reverse overlap primer to amplify the upstream region of the *vapC4* (*saci_1813*) gene | The present study |
| 13908 | 5’-TAGGGAAAGTCGTTGACCGTAGAGACAAGAA CAATCACAA AAC-3’ Forward overlap primer to amplify the downstream region of the *vapC4* (*saci_1813*) gene | The present study |
| 13909 | 5’-GGGAATTCCATATGCACTATCAACCGCAACCGATT TCAG-3’ Reverse primer to amplify the downstream region of the *vapC4* (*saci_1813*) gene (NdeI) | The present study |

**Table S2:** Strains used in the study

| **Strains** | **Relevant characteristics** | **Source of reference** |
| --- | --- | --- |
| *E. coli* XLIBlue | recA1 endA1 gyrA96 thi-1 hsdR17 supE44 relA1 lac [F’ proAB lacIq Z∆M15 Tn10 (Tet^r^)]Son | Stratagene |
| *E. coli* BL21 (DE3)-RIL | B F-ompT hsdS(rB-mB-) dcm+ Tet^r^ E. coli gal λ (DE3) endA Hte [argU ileY leuW Cam^r^] | Stratagene |
| *Sulfolobus acidocaldarius* DSM639 | Thermoacidophilic crenarchaeon which grows optimally at 75℃ and pH 2-3  WT | DSMZ |
| *Sulfolobus acidocaldarius* MW2000 | Thermoacidophilic crenarchaeon which grows optimally at 75℃ and pH 2-3  Δ*pyrEF* (91-412 bp) | Prof. Dr. Sonja-Verena Albers |
| *Sulfolobus acidocaldarius* MW1363 | Δ*pyrEF* (91-412 bp) Δ*vapC4* | The present study |
| *Sulfolobus acidocaldarius* MW1365 | Δ*pyrEF* (91-412 bp) Δ*vapBC4* | The present study |

Tet^r^, tetracycline resistant; Cam^r^, chloramphenicol resistant

**Table S3:** Plasmids used in the study

| **Plasmids** | **Relevant characteristics** | **Source of reference** |
| --- | --- | --- |
| pET28a | Kan^r^, Bacterial expression vector with T7 lac promoter containing replicon f1 and one MCS. | Novagen |
| pGEM3z | Amp^r^, bacterial transcription vector with T7 RNA polymerase promoter and SP6 promoter, replicon ColE1 (pUC18), and one MCS | Promega |
| pETDuet1 | Amp^r^, Bacterial expression vector with T7 lac promoter containing replicon ColE1 (pBR322) and two MCS. | Novagen |
| pSVA431 | Backbone for in-frame gene deletion in *S. acidocaldarius* | Wagner et. al. 2012 |
| pSVAaraFXStop | Backbone for complementing gene expression | Wagner et. al. 2012 |
| pAG153 | *vapC4 (saci_1813)* cloned into pET28a with C- terminal His tag | The present study |
| pAG154 | *vapB4 (saci_1812)* cloned into pET28a with C- terminal His tag | The present study |
| pAG155 | *vapBC4* operon cloned into pET28a with C-terminal His tag | The present study |
| pAG156 | *vapB4 (saci_1812)* cloned into pETDuet1 with C- terminal StrepII tag | The present study |
| pAG157 | *tRNA^Met^* gene cloned into pGEM3z vector for In-Vitro Transcription | The present study |
| pAG158 | 7S SRP-RNA gene cloned into pGEM3z vector for In-Vitro Transcription | The present study |
| pAG159 | 16S rRNA gene cloned into pGEM3z vector for In-Vitro Transcription | The present study |
| pAG160 | 23S rRNA gene cloned into pGEM3z vector for In-Vitro Transcription | The present study |
| pAG161 | *tetR* mRNA gene cloned into pGEM3z vector for In-Vitro Transcription | The present study |
| pAG162 | *saci_1223* mRNA gene into pGEM3z vector for In-Vitro Transcription | The present study |
| pAG163 | *saci_1242* mRNA gene cloned into pGEM3z vector for In-Vitro Transcription | The present study |
| pAG166 | *tRNA^Ser^* gene cloned into pGEM3z vector for In-Vitro Transcription | The present study |
| pAG167 | *tRNA^Pro^* gene cloned into pGEM3z vector for In-Vitro Transcription | The present study |
| pSVA6567 | KO plasmid Δ*vapBC4* cloned by overlap PCR and restrictions | The present study |
| pSVA6520 | KO plasmid Δ*vapC4* cloned by overlap PCR and restrictions | The present study |
| pSVA6523 | Expression of *vapB4* *saci_1812*, with 150 bps promoter (complementation) | The present study |
| pSVA6524 | Expression of *saci_1813* (premature stop codon on *saci_1812*), with 150 bps promoter (complementation) | The present study |
| pSVA6525 | Expression of *vapBC4* operon (*saci_1812-1813)* with 150 bps promoter (complementation) | The present study |

**Table S4:** The list of organisms whose genomes were used to perform phylogenomic analysis

| **Assembly Accession** | **Assembly Submitter** | **Organism Name** | **Assembly Stats GC Percent** | **Assembly Stats Total Sequence Length** |
| --- | --- | --- | --- | --- |
| GCA_009729015.1 | North Carolina State University | *Acidianus ambivalens* | 34 | 2252027 |
| GCA_003201835.2 | North Carolina State University | *Acidianus brierleyi* | 32 | 2947244 |
| GCA_002116695.1 | Central South University, China | *Acidianus manzaensis* | 30.5 | 2687463 |
| GCA_000591035.1 | Laboratory of Marine Microbiology, Division of Applied Biosciences, Graduate School of Agriculture, Kyoto University | *Aeropyrum camini SY1 = JCM 12091* | 56.5 | 1595994 |
| GCA_019058495.1 | The University of Queensland | Asgard group archaeon | 46.5 | 2881860 |
| GCA_026993975.1 | Portland State University | Asgard group archaeon | 36 | 2303441 |
| GCA_027016435.1 | Portland State University | Asgard group archaeon | 36.5 | 1250612 |
| GCA_000270325.1 | Japan Agency for Maine-Earth Science and Technology | *Candidatus Caldarchaeum subterraneum* | 51.5 | 1680938 |
| GCA_026997665.1 | Portland State University | *Candidatus Heimdallarchaeota archaeon* | 43 | 2066261 |
| GCA_026999965.1 | Portland State University | *Candidatus Heimdallarchaeota archaeon* | 37 | 1821052 |
| GCA_027018025.1 | Portland State University | *Candidatus Heimdallarchaeota archaeon* | 42 | 4628237 |
| GCA_027054495.1 | Portland State University | *Candidatus Heimdallarchaeota archaeon* | 37 | 3878664 |
| GCA_027334505.1 | An eLibrary of Microbial Systematics and Genomics | *Candidatus Heimdallarchaeota archaeon* | 29.5 | 4203295 |
| GCA_002214165.1 | Fidelity Systems | *Candidatus Mancarchaeum acidiphilum* | 39.5 | 952257 |
| GCA_905171695.1 | Max Planck Institute for Marine Microbiology | *Candidatus Methanoperedenaceae archaeon GB37* | 46.5 | 1922745 |
| GCA_905171685.1 | Max Planck Institute for Marine Microbiology | *Candidatus Methanoperedenaceae archaeon GB50* | 46.5 | 1920273 |
| GCA_000730285.1 | University of Florida | *Candidatus Nitrososphaera evergladensis SR1* | 50 | 2954373 |
| GCA_000303155.1 | Georg-August-University Gottingen | *Candidatus Nitrososphaera gargensis Ga9.2* | 48.5 | 2833868 |
| GCA_013340765.1 | Japan Collection of Microorganisms | *Conexivisphaera calida* | 62 | 1593902 |
| GCA_000152265.2 | DOE Joint Genome Institute | *Ferroplasma acidarmanus Fer1* | 36.5 | 1935211 |
| GCA_002078355.1 | Max Planck Institute for Chemical Ecology | *Ferroplasma acidiphilum* | 36.5 | 1826943 |
| GCA_000258425.1 | Centre Bioengineering RAS | *Fervidicoccus fontis Kam940* | 37.5 | 1319206 |
| GCF_020618475.1 | Jiangsu University | *Haladaptatus halobius* | 57.5 | 7279389 |
| GCA_010092465.1 | AMAAS | *haloarchaeon 3A1-DGR* | 67.5 | 2890613 |
| GCA_000015145.1 | Univ. Copenhagen | *Hyperthermus butylicus DSM 5456* | 53.5 | 1667163 |
| GCA_000145985.1 | US DOE Joint Genome Institute (JGI-PGF) | *Ignisphaera aggregans DSM 17230* | 35.5 | 1875953 |
| GCA_014876775.1 | Winogradsky Institute of Microbiology | *Infirmifilum lucidum* | 54 | 1625846 |
| GCA_000993805.1 | Immanuel Kant Baltic Federal University | *Infirmifilum uzonense* | 48 | 1611988 |
| GCA_021654415.1 | Department of Science and Engineering for Sustainable Development, Faculty of Science and Engineering, Soka University | *Metallosphaera javensis* | 47 | 2464819 |
| GCF_005222525.1 | North Carolina State University | *Metallosphaera prunae* | 46 | 2202576 |
| GCA_001266655.1 | University of Nebraska-Lincoln | *Metallosphaera sedula* | 46 | 2191533 |
| GCA_001266675.1 | University of Nebraska-Lincoln | *Metallosphaera sedula* | 46 | 2191529 |
| GCA_001266695.1 | University of Nebraska-Lincoln | *Metallosphaera sedula* | 46 | 2191531 |
| GCA_001266715.1 | University of Nebraska-Lincoln | *Metallosphaera sedula* | 46 | 2191533 |
| GCA_001266735.1 | University of Nebraska-Lincoln | *Metallosphaera sedula* | 46 | 2191533 |
| GCA_000204415.1 | University of Wisconsin-Parkside | *Methanothrix soehngenii GP6* | 51 | 3026645 |
| GCF_013402815.2 | Jiangsu University | *Natrinema halophilum* | 60 | 4842193 |
| GCA_000328685.1 | JGI | *Natronococcus occultus SP4* | 64.5 | 4314118 |
| GCA_000234805.1 | Centre Bioengineering RAS | *Pyrobaculum ferrireducens* | 57 | 2467972 |
| GCA_000015205.1 | DOE Joint Genome Institute | *Pyrobaculum islandicum DSM 4184* | 49.5 | 1826402 |
| GCA_001189275.1 | Montana State University | *Pyrobaculum sp. WP30* | 58.5 | 1993257 |
| GCA_001412615.1 | Kyung Hee University | *Pyrodictium delaneyi* | 54 | 2023836 |
| GCA_000223395.1 | DOE Joint Genome Institute | *Pyrolobus fumarii 1A* | 55 | 1843267 |
| GCA_019175305.1 | Skolkovo Institute of Science and Technology | *Saccharolobus shibatae* | 35.5 | 2791785 |
| GCA_019175325.1 | Skolkovo Institute of Science and Technology | *Saccharolobus shibatae* | 35.5 | 2778607 |
| GCA_019175345.1 | Skolkovo Institute of Science and Technology | *Saccharolobus shibatae B12* | 35.5 | 2916875 |
| GCF_019693215.1 | Chinese academy of science | *Salinarchaeum sp. IM2453* | 47.5 | 2656678 |
| GCA_000092465.1 | US DOE Joint Genome Institute (JGI-PGF) | *Staphylothermus hellenicus DSM 12710* | 37 | 1580347 |
| GCA_000015945.1 | US DOE Joint Genome Institute | *Staphylothermus marinus F1* | 35.5 | 1570485 |
| GCA_003967175.1 | Laboratory of Extremophiles, Environmental Engineering for Symbiosis, Graduate school Soka University | *Sulfodiicoccus acidiphilus* | 51 | 2353189 |
| GCF_002215565.1 | Harvard University | *Sulfolobus acidocaldarius* | 36.5 | 2225849 |
| GCA_002215405.1 | Harvard University | *Sulfolobus acidocaldarius* | 36.5 | 2171591 |
| GCA_002215445.1 | Harvard University | *Sulfolobus acidocaldarius* | 36.5 | 2270336 |
| GCA_002215485.1 | Harvard University | *Sulfolobus acidocaldarius* | 37 | 2286709 |
| GCA_002215525.1 | Harvard University | *Sulfolobus acidocaldarius* | 36.5 | 2238200 |
| GCA_000012285.1 | Danish Archaea Centre | *Sulfolobus acidocaldarius DSM 639* | 36.5 | 2225959 |
| GCA_000340315.1 | University of Cincinnati | *Sulfolobus acidocaldarius N8* | 36.5 | 2176362 |
| GCF_000338775.1 | University of Cincinnati | *Sulfolobus acidocaldarius Ron12/I* | 36.5 | 2223983 |
| GCA_000508305.1 | Center for Genomic Sciences | *Sulfolobus acidocaldarius SUSAZ* | 36.5 | 2061920 |
| GCF_028472005.1 | Wuhan Unverisity | *Sulfolobus islandicus* | 35 | 2656591 |
| GCF_000024305.1 | US DOE Joint Genome Institute | *Sulfolobus islandicus L.D.8.5* | 35.5 | 2748647 |
| GCF_000364745.1 | Pasteur Institute, Paris, France | *Sulfolobus islandicus LAL14/1* | 35 | 2465177 |
| GCF_000189555.1 | State Key Laboratory of Microbial Resources, Institute of Microbiology, CAS, Beijing, P. R. China | *Sulfolobus islandicus REY15A* | 35.5 | 2522992 |
| GCA_000022465.1 | US DOE Joint Genome Institute | *Sulfolobus islandicus Y.G.57.14* | 35.5 | 2702058 |
| GCF_000022485.1 | US DOE Joint Genome Institute (JGI-PGF) | *Sulfolobus islandicus Y.N.15.51* | 35.5 | 2854410 |
| GCF_001719125.1 | Institute of Microbiology, Chinese Academy of Sciences | *Sulfolobus sp. A20* | 35 | 2688317 |
| GCA_008326385.1 | RIKEN-BRC | *Sulfuracidifex tepidarius* | 42.5 | 2535282 |
| GCA_008326425.1 | RIKEN-BRC | *Sulfuracidifex tepidarius* | 42.5 | 2560765 |
| GCF_000585495.1 | Institute de Genetique et Microbiologie | *Thermococcus nautili* | 55 | 1976356 |
| GCA_002214505.1 | Ecole Normale Superieure de Lyon | *Thermococcus siculi* | 55 | 2029167 |
| GCA_000221185.1 | Key Laboratory of Marine Biogenetic Resources, Third Institute of Oceanography, State Oceanic Administration (SOA),PR China | *Thermococcus sp. 4557* | 56 | 2011320 |
| GCA_000151205.2 | Moore Foundation | *Thermococcus sp. AM4* | 55 | 2086428 |
| GCA_000446015.1 | Baltic Federal University | *Thermofilum adornatum* | 46.5 | 1750259 |
| GCF_000813245.1 | Centre Bioengineering RAS | *Thermofilum adornatum 1505* | 46.5 | 1754190 |
| GCA_000015225.1 | DOE Joint Genome Institute | *Thermofilum pendens Hrk 5* | 57.5 | 1813393 |
| GCA_000264495.1 | Centre Bioengineering RAS | *Thermogladius calderae 1633* | 55.5 | 1356318 |
| GCA_016806715.1 | Karlsruhe Institute of Technology | *Thermoplasmatales archaeon* | 44.5 | 1959588 |
| GCA_000148385.1 | US DOE Joint Genome Institute (JGI-PGF) | *Vulcanisaeta distributa DSM 14429* | 45.5 | 2374137 |

**Table S5:** Description of the putative 25 pairs homologous genes of the toxin-antitoxin system

| **Organisms** | **Superphylum** | ***saci_1813* gene annotation** | ***saci_1812* gene annotation** |
| --- | --- | --- | --- |
| Asgard_group_archaeon_GCA_019058495.1 | Asgardarchaeota | Exonuclease VapC9 | hypothetical protein |
| Asgard_group_archaeon_GCA_027016435.1 | Asgardarchaeota | Ribonuclease VapC3 | hypothetical protein |
| *Candidatus_Heimdallarchaeota_archaeon_GCA_026997665.1* | Asgardarchaeota | Ribonuclease VapC3 | hypothetical protein |
| *Candidatus_Mancarchaeum_acidiphilum_Mia14* | DPANN | Ribonuclease VapC3 | hypothetical protein |
| *Candidatus_Methanoperedenaceae_archaeon_GB37_Arc1_E37* | Euryarchaeota | Ribonuclease VapC3 | hypothetical protein |
| *Candidatus_Methanoperedenaceae_archaeon_GB50_ Arc1_E50* | Euryarchaeota | Ribonuclease VapC3 | hypothetical protein |
| *Haladaptatus_sp_PSR5_PSR5* | Euryarchaeota | hypothetical protein | hypothetical protein |
| *Ignisphaera_aggregans_DSM_17230_DSM_17230* | TACK | Exonuclease VapC9 | hypothetical protein |
| *Infirmifilum_lucidum_3507LT* | TACK | Exonuclease VapC9 | hypothetical protein |
| *Natronococcus_occultus_SP4_SP4* | Euryarchaeota | Exonuclease VapC9 | hypothetical protein |
| *Pyrobaculum_islandicum_DSM_4184_DSM_4184* | TACK | Ribonuclease VapC3 | hypothetical protein |
| *Staphylothermus_hellenicus_DSM_12710_DSM_12710* | TACK | Exonuclease VapC9 | hypothetical protein |
| *Sulfolobus_acidocaldarius_DG1* | TACK | Exonuclease VapC9 | hypothetical protein |
| *Sulfolobus_acidocaldarius_DSM_639* | TACK | Exonuclease VapC9 | hypothetical protein |
| *Sulfolobus_acidocaldarius_N8_N8* | TACK | Exonuclease VapC9 | hypothetical protein |
| *Sulfolobus_acidocaldarius_Ron12_I_Ron12_I* | TACK | Exonuclease VapC9 | hypothetical protein |
| *Sulfolobus_acidocaldarius_SUSAZ_SUSAZ* | TACK | Exonuclease VapC9 | hypothetical protein |
| *Sulfolobus_acidocaldarius_Y14_13-1* | TACK | Exonuclease VapC9 | hypothetical protein |
| *Sulfolobus_acidocaldarius_Y14_16-22* | TACK | Exonuclease VapC9 | hypothetical protein |
| *Sulfolobus_acidocaldarius_Y14_18-5* | TACK | Exonuclease VapC9 | hypothetical protein |
| *Sulfolobus_acidocaldarius_Y14_20-20* | TACK | Exonuclease VapC9 | hypothetical protein |
| *Sulfolobus_sp_A20_A20* | TACK | hypothetical protein | hypothetical protein |
| *Thermococcus_nautili_30-1* | Euryarchaeota | Ribonuclease VapC3 | hypothetical protein |
| *Thermococcus_sp_AM4_AM4* | Euryarchaeota | Ribonuclease VapC3 | hypothetical protein |
| *Thermofilum_pendens_Hrk_5_Hrk_5* | TACK | Ribonuclease VapC3 | hypothetical protein |

**
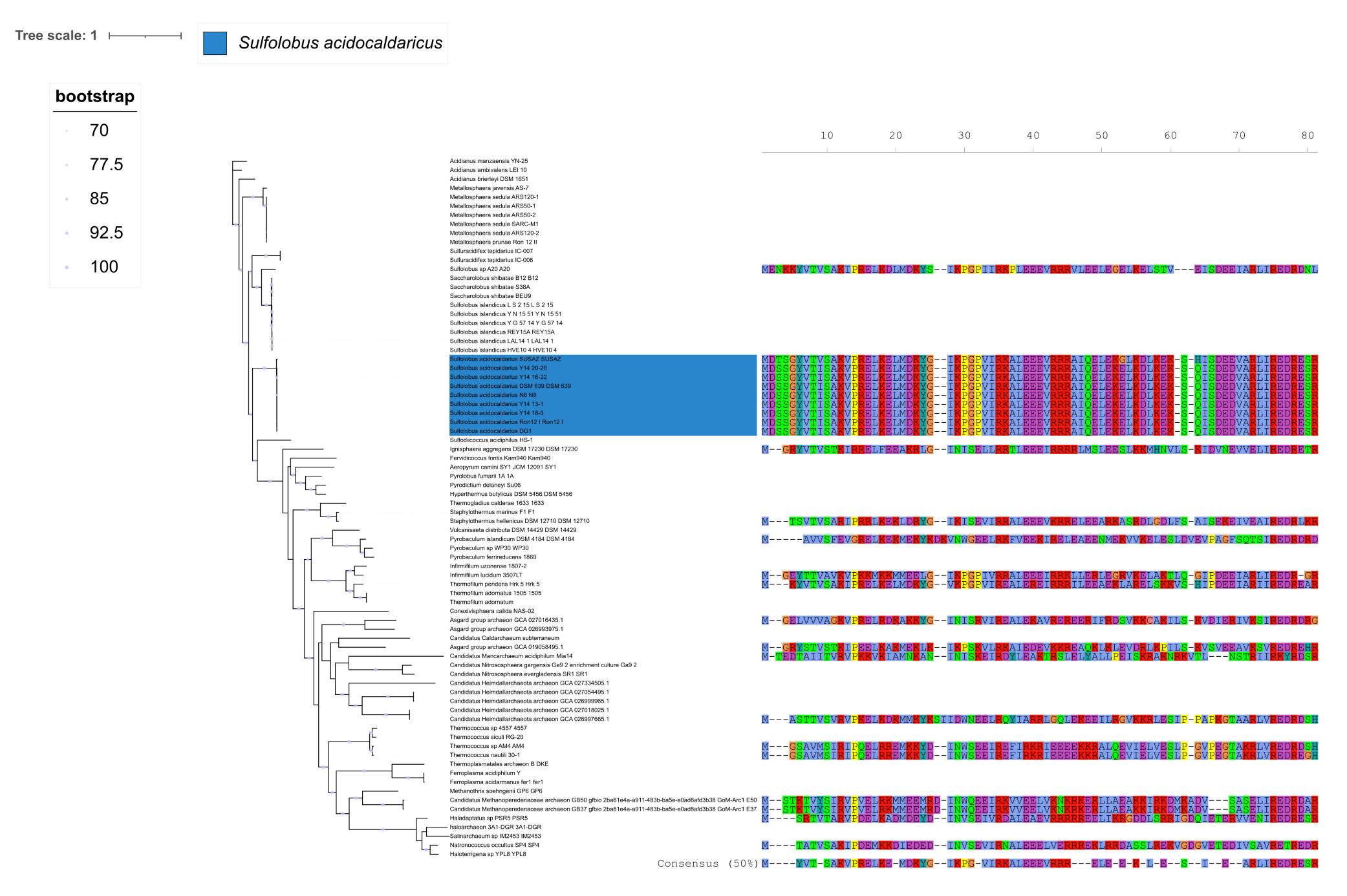
Figure S1:** Phylogenetic perspective of the 25 homologues of the *vapB4* (*saci_1812*) gene. The leaves of the tree that correspond to lineages of organisms. The leaves of the tree that corresponds to lineage of the genus *Sulfolobus acidocaldarius* are highlighted in blue

**Figure S2:** Phylogenetic perspective of the 25 homologues of the *vapC4* (*saci_1813*) gene. The leaves of the tree correspond to lineages of organisms. The leaves of the tree that corresponds to lineage of the genus *Sulfolobus acidocaldarius* are highlighted in blue

**
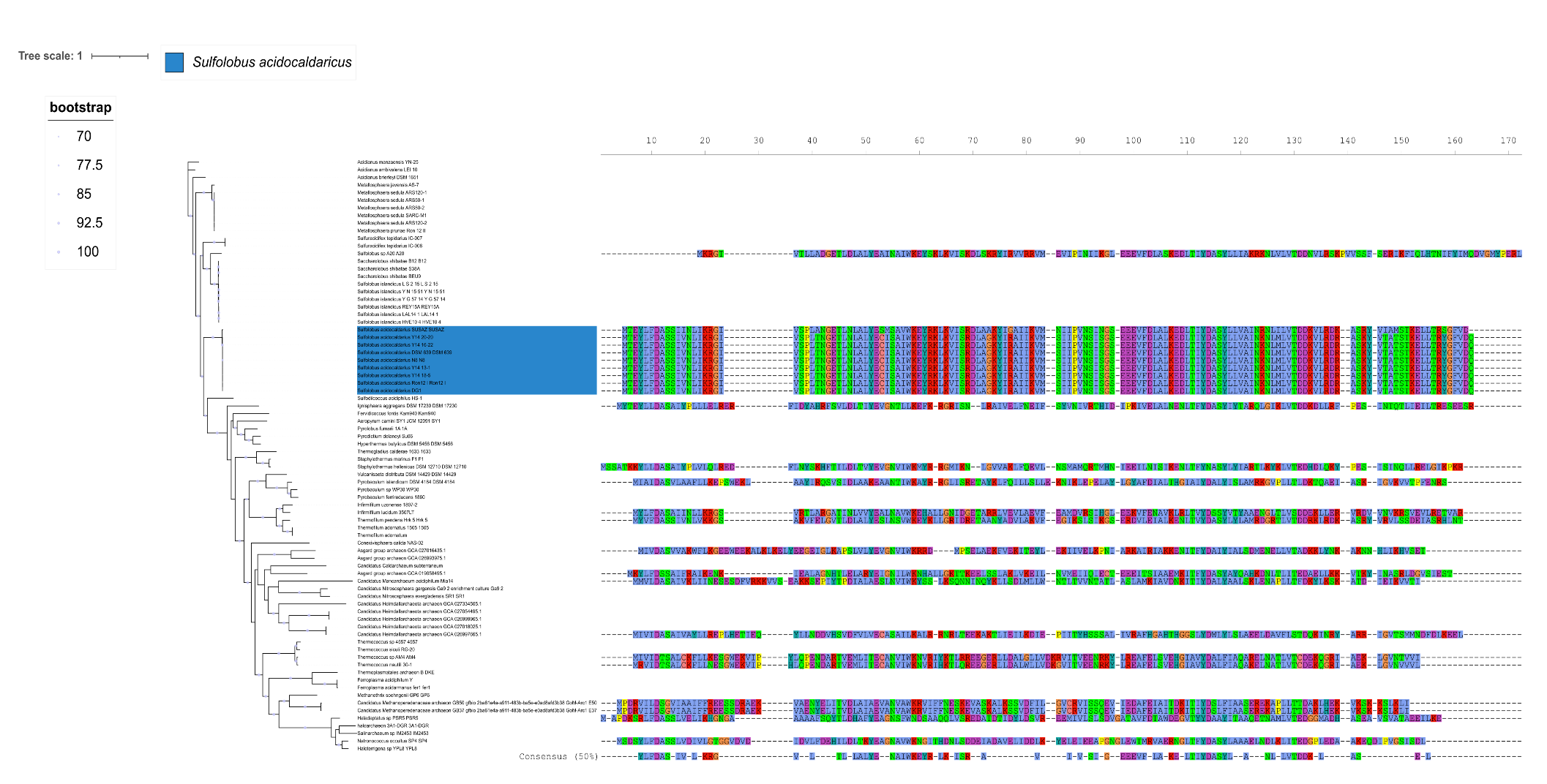
**

**Figure S3:** Western blot analysis of the Elution 2 fraction obtained after Ni-NTA affinity chromatography of VapC4 and VapB4 proteins using an anti-His antibody.

**
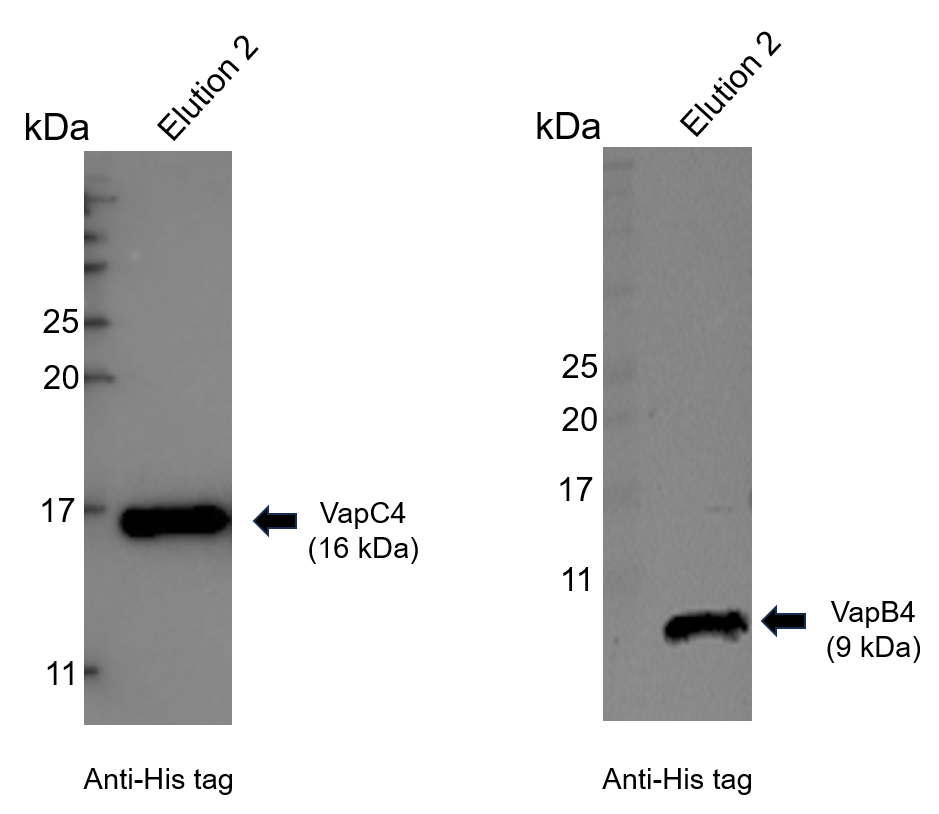
**

**Figure S4:** Graph showing the percentage of RNA following densitometric analysis of 23S rRNA and 16S rRNA bands from total RNA extracted from *Sulfolobus acidocaldarius* after exposure to increasing concentrations of the VapC4 toxin protein at two different temperatures (37°C and 60°C). The statistical significance, P < 0.05, is indicated as *, P < 0.01 is indicated as **, and P < 0.001 is indicated as ***; n.s.denotes not significant.

**
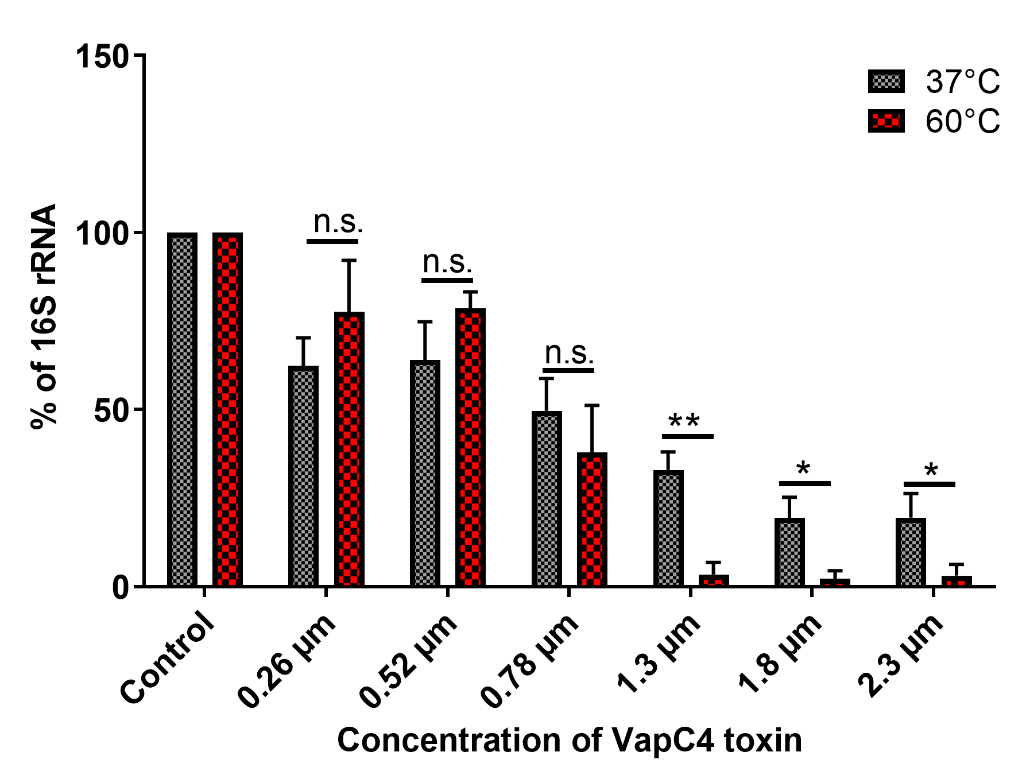

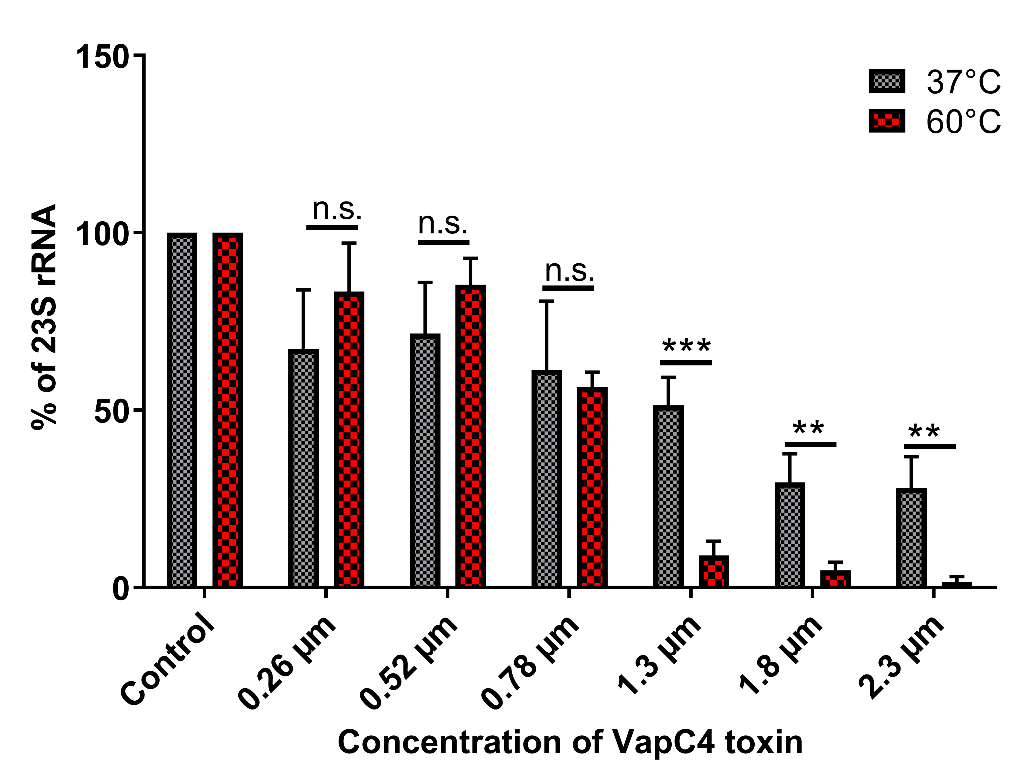
**

**Figure S5:** 1% Agarose gel showing RNase activity of VapC4 on different RNA substrates: a) tRNA^Ser^ b) tRNA^Pro^ c) *saci_1223* mRNA transcript and *saci_1242* mRNA transcript.

**
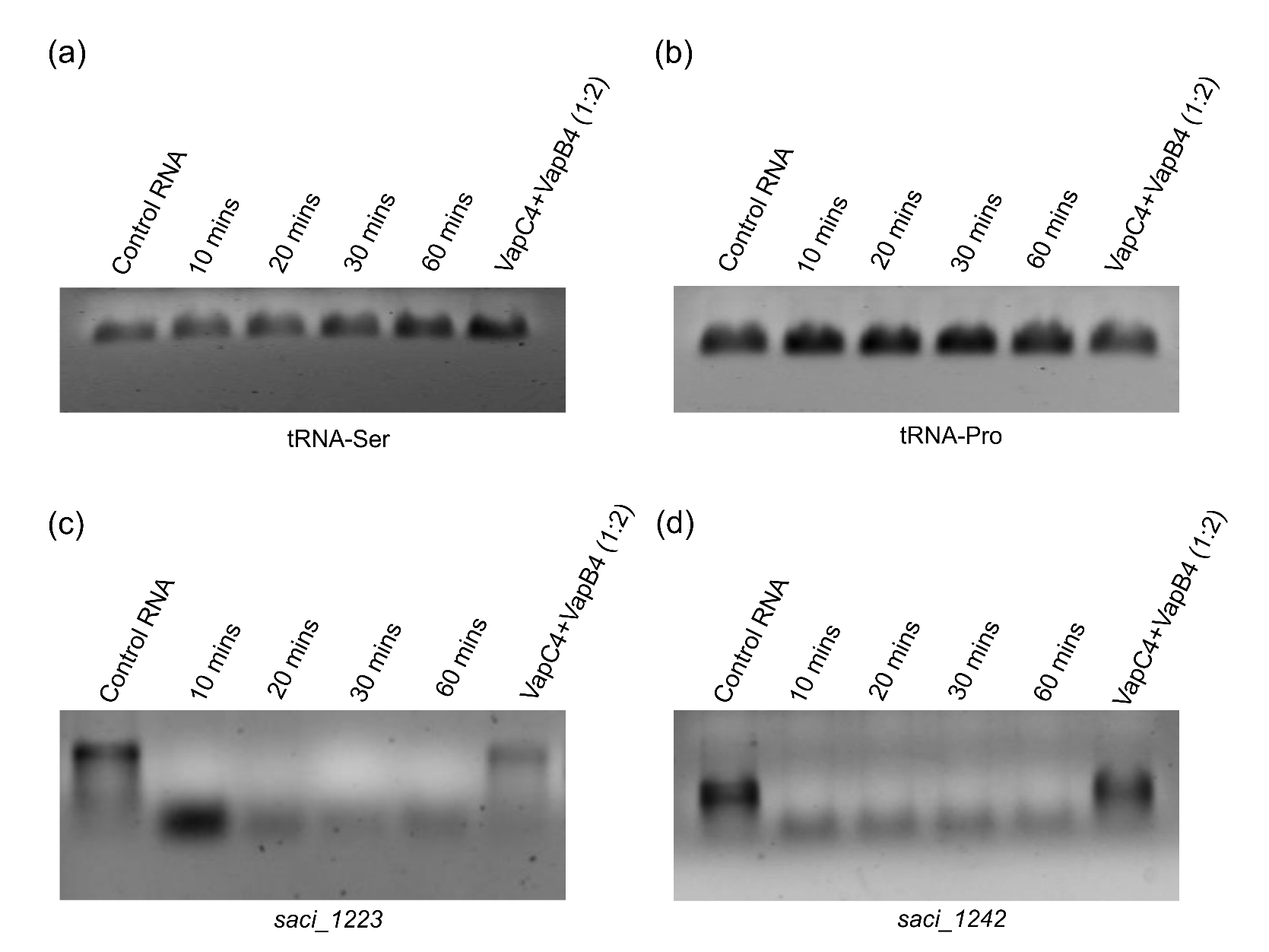
**

**Figure S6:** A 15% SDS-PAGE analysis revealed the absence of VapB4 antitoxin elution (at the 9 kDa position) in the Elution fraction when *E. coli* cells expressing solely the VapB4 antitoxin with a Strep-II tag underwent Ni-NTA affinity chromatography


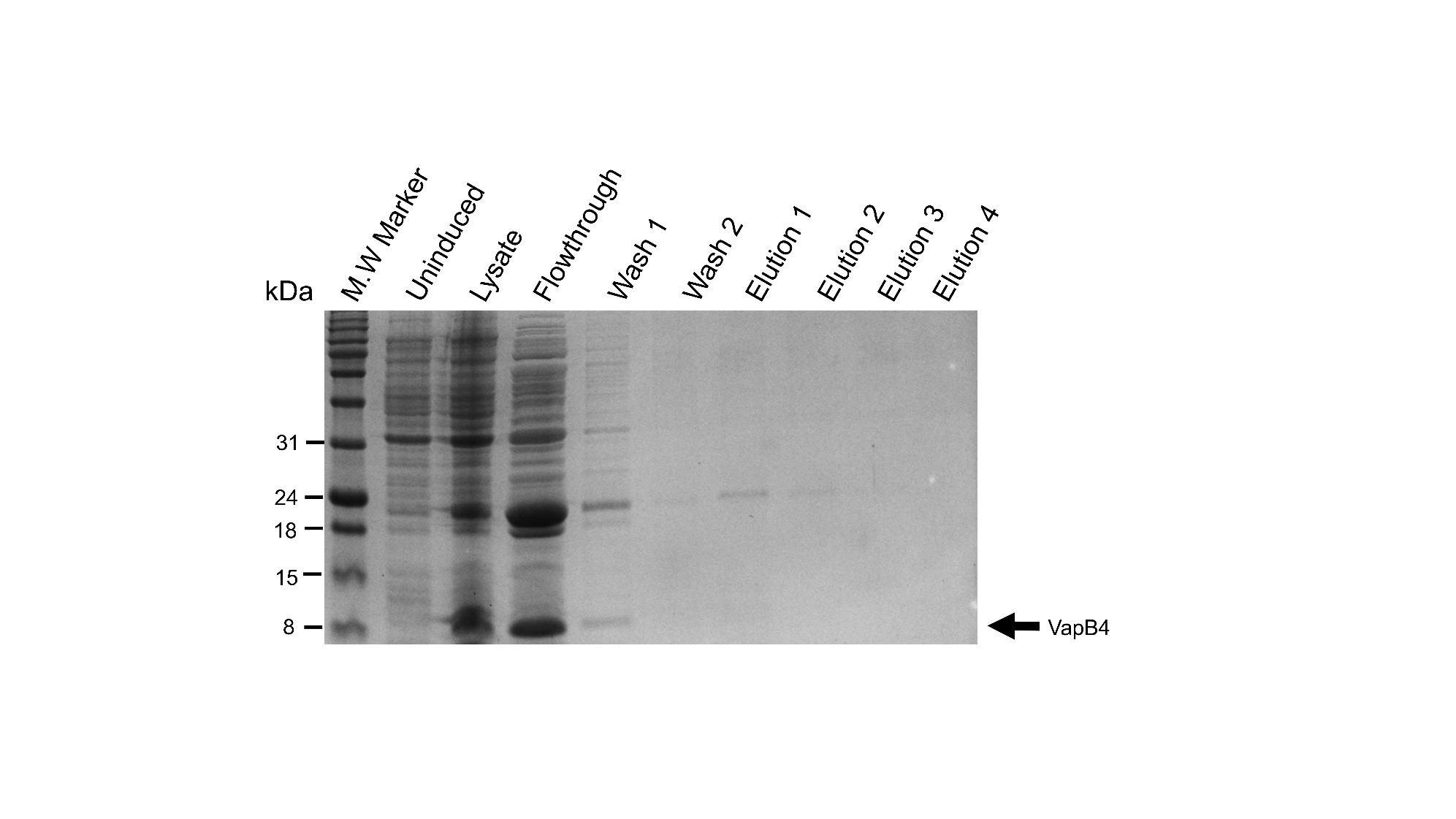


**Figure S7**: Agarose gel showing PCR amplification products to verify the absence of *vapC4* and *vapBC4* genes in the Δ*vapC4* and Δ*vapBC4* strains of *S. acidocaldarius* MW2000 and to check the status of *vapC4*, *vapB4* and *vapBC4* genes in the generated complementation strain using *vapC4*, *vapB4* and *vapBC4* gene-specific primers, respectively


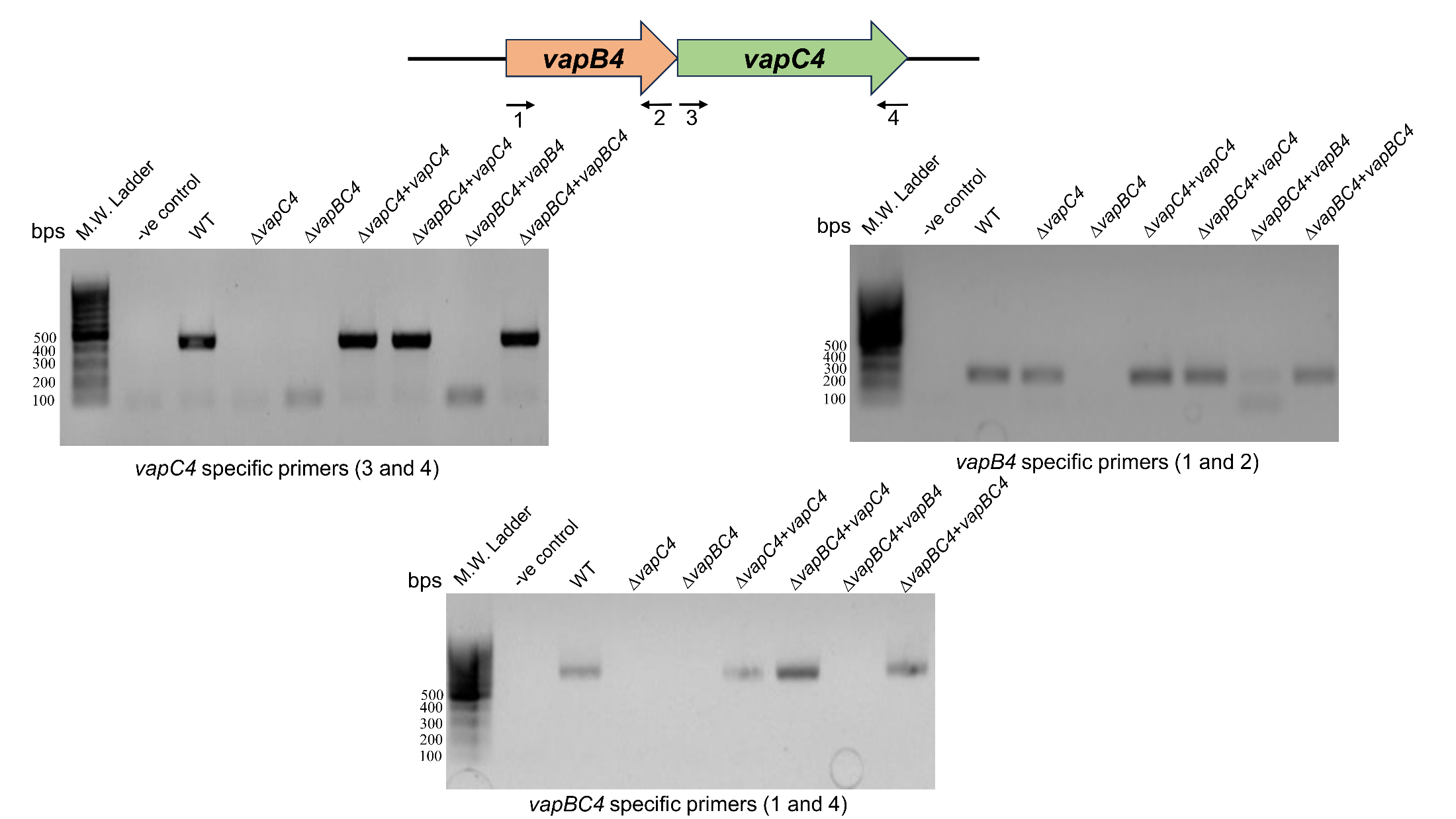

Supplement: Supplemental material — Supplemental tables and figures. [file mbio.02753-24-s0001.docx]
